# Management of ruptured aneurysmal subarachnoid hemorrhage with multiple basilar trunk aneurysms using a flow-diverter stent: A case report

**DOI:** 10.1016/j.radcr.2024.03.038

**Published:** 2024-04-13

**Authors:** Mai D. Ton, Dao V. Phuong, Pham Q. Tho, Nguyen T. Dung, Tran A. Tuan, Nguyen T. Thien

**Affiliations:** a Stroke Center, Bach Mai Hospital, 78 Giai Phong Street, Dong Da District, Hanoi, Vietnam; b Hanoi Medical University, No 1, Ton That Tung Street, Dong Da District, Hanoi, Vietnam; c VNU University of Medicine and Pharmacy; 144 Xuan Thuy Street, Cau Giay District, Hanoi, Vietnam; d Radiology Center, Bach Mai Hospital, 78 Giai Phong Street, Dong Da District, Hanoi, Vietnam

**Keywords:** Subarachnoid hemorrhage, Multiple basilar trunk aneurysms, Flow diverter stent

## Abstract

Ruptured aneurysmal subarachnoid hemorrhage associated with multiple basilar trunk aneurysms represents a rare clinical condition. Endovascular intervention stands as the preferred therapeutic approach. We present the case of a 35-year-old patient with subarachnoid hemorrhage and three consecutive basilar trunk aneurysms. Utilizing a flow-diverter stent, we achieved simultaneous occlusion of all 3 aneurysms, performed 2 hours post dual antiplatelet therapy (comprising salicylic acid 300 mg and ticagrelor 180 mg). Sustained resistance to clopidogrel necessitated the subsequent 3 months, followed by single antiplatelet therapy. At the 1-month follow-up, the patient demonstrated a favorable clinical course, devoid of cerebral infarction, and evidenced unobstructed stent patency upon brain magnetic resonance imaging.

## Introduction

Subarachnoid hemorrhage (SAH) resulting from ruptured intracranial aneurysms (IA) is associated with a poor prognosis and heightened mortality risk [1]. Rebleeding, particularly within the initial 24 hours, stands as a main cause of mortality. Timely and secure treatment for IA is imperative, ideally within 24 hours of onset [2].

Multiple intracranial aneurysms (MIAs), which are not a rare condition, account for up to 30% of IA patients [3], the occurrence of multiple basilar trunk aneurysms—defined as aneurysms located between the origin of the basilar artery and below the superior cerebellar artery—is relatively infrequent, with limited reports pertaining to SAH patients with multiple basilar trunk aneurysms [4].

We present a case involving an SAH patient afflicted with 3 consecutive basilar trunk aneurysms, alongside clopidogrel resistance, successfully managed using a flow-diverter stent.

## Case report

**Fig. 1:**
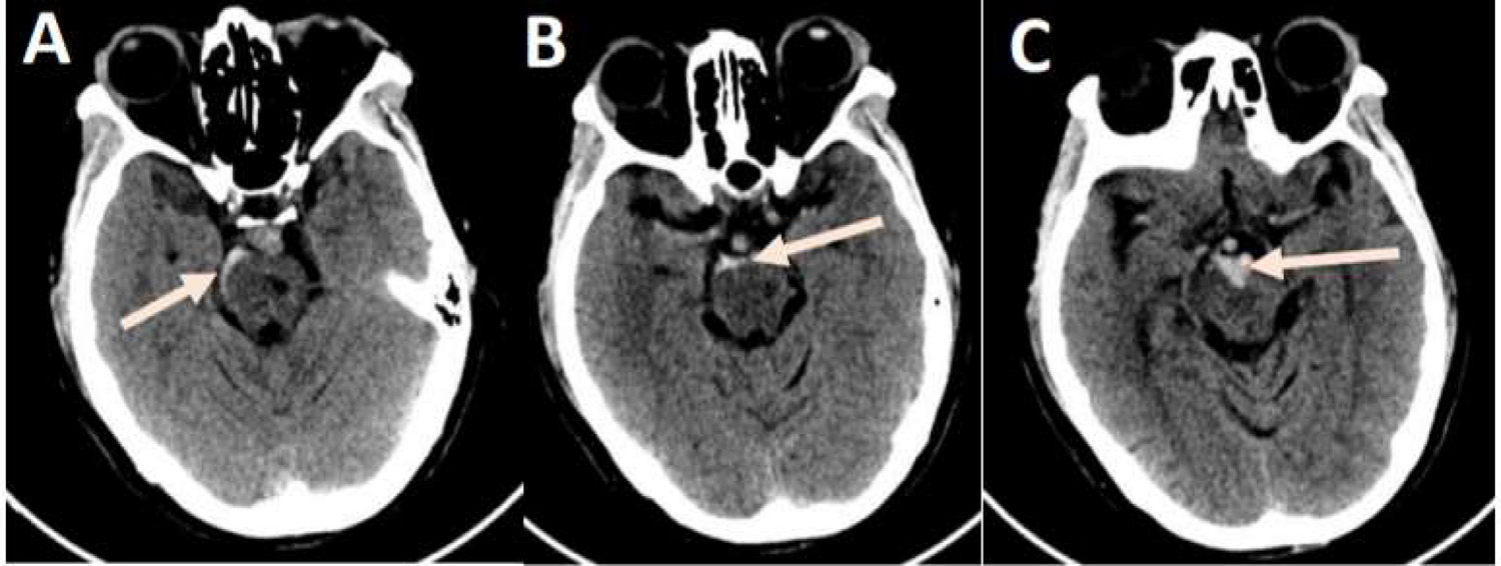
Subarachnoid hemorrhage around the pons and brain stem (red arrows).

A 35-year-old female patient was admitted to the hospital due to the onset of a severe headache on the second day. The patient, with no prior prescriptions and no history of injury or falls, presented with a severe headache but had not exhibited seizures or focal neurological signs the previous day. Following a clinical examination at a local hospital, the patient was diagnosed with SAH and subsequently transferred to a specialized stroke center with a Glasgow coma scale score of 15 points, stiff neck, a positive Kernig sign, blood pressure measuring 150/70 mmHg, and a pulse rate of 80 beats per minute. CT scanner revealed subarachnoid hemorrhage surrounding the pons and brain stem (Fig. 1).

**Fig. 2:**
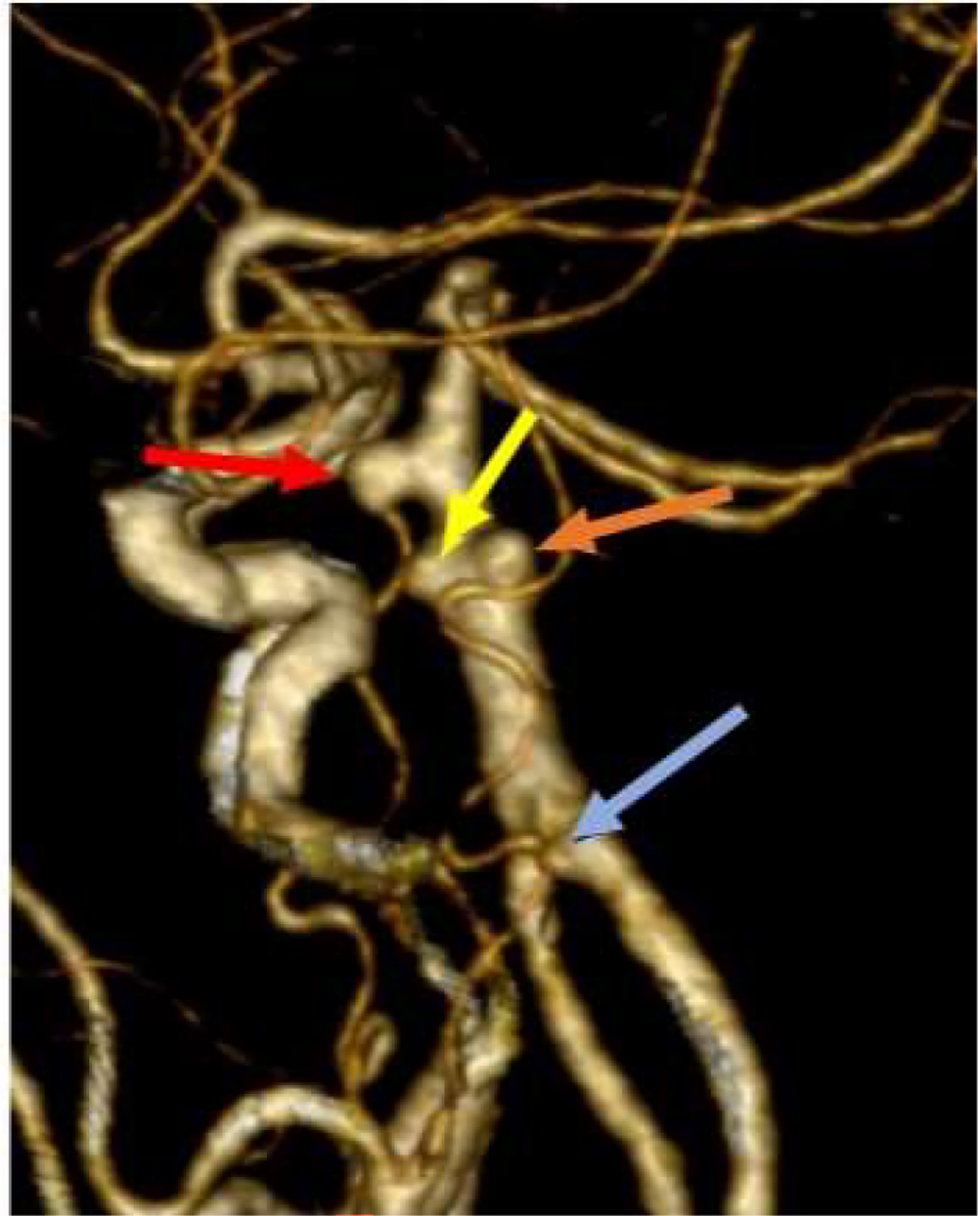
Multiple basilar trunk aneurysms. Red, yellow, and orange arrows indicated basilar trunk aneurysms, whereas blue one indicated small vertebral aneurysm.

Cerebral angiography pointed out 3 wide-necked basilar trunk aneurysms, sizes 4.3*5.6, 2.4*4.6 and 4.1*5.3, respectively **(**Fig. 2**)**. The three aneurysms were situated in close proximity to one another, positioned superior to the anterior inferior cerebellar artery (AICA) and inferior to the superior cerebellar artery (SCA). Additionally, a small aneurysm (2.4*3 mm) was identified in the left vertebral artery, distant from the site of hemorrhage.

A multidisciplinary consultation involving a stroke physician, interventional radiologist, and neurosurgeon was conducted, leading to the recommendation of employing a flow-diverter stent to address all 3 basilar trunk aneurysms. To ensure the desired antiplatelet effect, administration of salicylic acid 300 mg and Ticagrelor 180 mg took place 2 hours prior to the intervention.

**Fig. 3:**
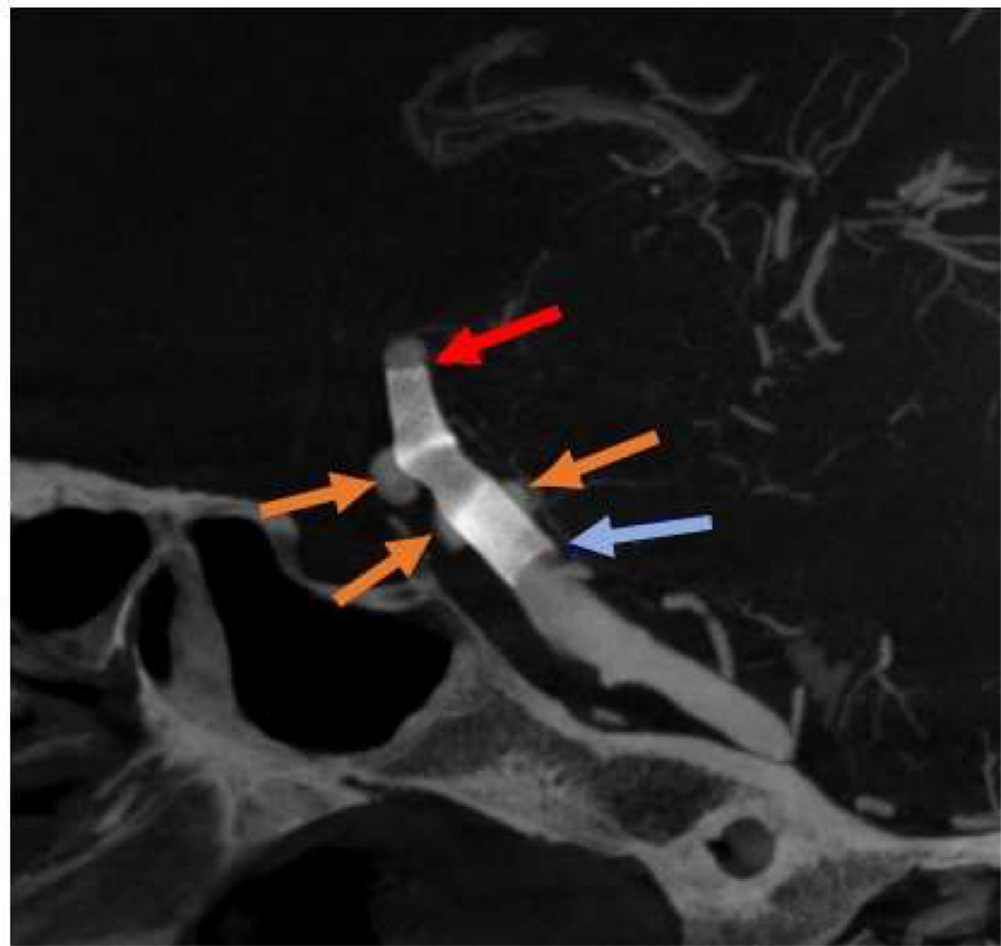
DSA image showed that stent covered all 3 aneurysms. Red and blue arrows indicated distal and proximal ends of the stent, respectively, whereas orange arrows indicated aneurysms.

Intervention procedure:

• The patient was intubated and placed under general anesthesia.
• Intervention was performed through right common femoral artery access. Sheath 8F, Porter 6F were used to approach the left vertebral artery and identify 3 aneurysms. Images of an unevenly dilated basilar artery with the largest diameter of 6.5 mm was noticed.
• Two catheter - NeuronMax 8F and Delivery 6F – were used to access the left vertebral artery, while Phenom27 microcatheter was used to access the right posterial cerebral artery, and deploy a 5/20 Pipeline stent covering all 3 aneurysms.
• Follow-up images were taken to ensure that the stent had been completely expanded and close to the vessel wall (Fig. 3).

**Fig. 4:**
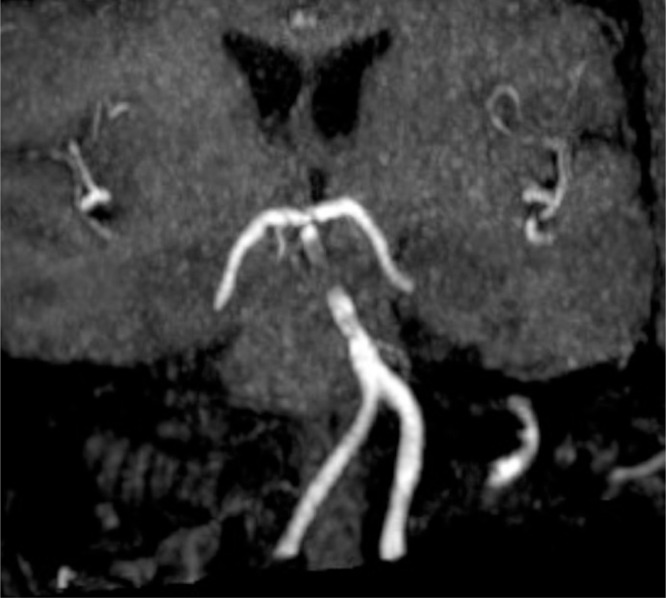
One-month follow-up MRI without obstruction within the stent.

The patient's ongoing treatment regimen included oral administration of Nimodipine 60 mg every 4 hours, along with salicylic acid 81 mg once daily and ticagrelor 90 mg twice daily, in addition to analgesics of the CYP2C19 gene, which demonstrated the presence of the CYP2C19 *2/*2 genotype, indicating the patient's homozygosity for 2 loss-of-function alleles. Consequently, the patient exhibited an impaired capacity to metabolize clopidogrel, classifying as a poor metabolizer. The patient was discharged after a 7-day hospitalization and subsequently maintained a dual antiplatelet therapy (DAPT) regimen, consisting of salicylic acid 81 mg once daily and ticagrelor 90 mg twice daily, for the ensuing 3 months. During the 1-month follow-up assessment, a brain magnetic resonance imaging scan was conducted, revealing an absence of infarction lesions and stent obstruction (Fig. 4)***.***

## Discussion

SAH resulting from a ruptured intracranial aneurysm represents a critical medical emergency, associated with a mortality rate of up to 16% among hospitalized patients [2]. Urgent intervention for a ruptured aneurysm is imperative to mitigate the risk of rebleeding, a complication that carries a mortality rate as high as 80% [5].

Approximately 30% of IA patients have multiple aneurysms (≥2 aneurysms) [6]. In cases of SAH where MIAs are identified, accurate identification of the causative aneurysm is paramount, as mismanagement of the ruptured source may result in grave consequences. Aneurysm characteristics, encompassing irregular shape, size, and location serve as pivotal indicators in discerning the culprit aneurysm [7].

Some methods are currently used to secure ruptured IA including clipping surgery and endovascular intervention (coiling, flow-diverter stenting, or stent-assisted coiling). Coiling is preferred for the treatment of saccular, narrow-neck aneurysms of posterior circulation, whereas flow-diverter stents may be used to treat patients who are not suitable for coiling or clipping (including blister-like and fusiform aneurysms) [2]. Recently, several studies show the effectiveness and safety of flow diverter stenting for SAH patients. A 2022 meta-analysis showed that among 357 SAH patients treated with flow diverter stenting, the rate of complete aneurysm occlusion was 85.6%, good clinical outcome was 73.7%, and the mortality rate was only 17.1% [8].

Our patient had bleeding around the pons and brainstem, accompanied by 3 high-risk basilar trunk aneurysms which located adjacent to each other. The percentage of multiple basilar trunk aneurysms, according to Saliou et al. is only about 2.1% [4]. However, exactly identifying the ruptured aneurysm in this patient, based on the location, shape, and direction of the aneurysm, is not highly reliable. In fact, some reports show that the culprit aneurysm cannot be accurately identified in about one-third of SAH patients with MIAs [6]. Therefore, the proposed solution is to simultaneously treat 3 aneurysms with basilar artery flow diverter stent.

It is noticeable that flow diverter treatment requires using of DAPT, which is challenging for clinicians because of increasing risk of rebleeding/progressive bleeding. In Vietnam, there are currently 3 groups of commonly used antiplatelet active ingredients: COX-1 inhibitors (salicylic acid), P2Y12 inhibitors (clopidogrel, ticagrelor), and PDE inhibitors (cilostazol). Salicylic acid and ticagrelor have a short time of action onset, average of 20-30 minutes, and are chosen in this case to reduce the risk of thromboembolic complications [9].

In addition, the clopidogrel resistance is a matter of significant concern. Clopidogrel, functioning as a prodrug, undergoes hepatic metabolism to form an active metabolite, which acts to inhibit platelet aggregation. In Hasan's study elucidates that CYP2C19 metabolic polymorphism contributes to a prevalent reduction in response to clopidogrel within Asian populations, potentially affecting up to 70% individuals in certain communities [10].

Our patient had homozygous CYP2C19 *2/*2 genotype test results indicating poor clopidogrel metabolism [11]. We decided to maintain DAPT regimen with salicylic acid and ticagrelor for a period of 3 months, followed by single antiplatelet therapy.

## Conclusion

Ruptured aneurysm SAH with multiple basilar trunk aneurysms represents an infrequent pathological entity. Endovascular intervention utilizing flow-diverter stents stands as a viable therapeutic approach warranting consideration.

Additionally, the assessment of CYP2C19 gene polymorphism is imperative for devising an optimal treatment regimen.

## Author contributions

All authors provided substantial contributions to the manuscript and approved the final version of the article to be published.

## Patient consent

An informed consent was obtained from the patient.
